# Sociodemographic and clinical features of postpartum depression among Turkish women: a prospective study

**DOI:** 10.1186/s12884-015-0532-1

**Published:** 2015-05-03

**Authors:** Ayse Figen Turkcapar, Nezaket Kadıoğlu, Ebru Aslan, Suphi Tunc, Müjdegül Zayıfoğlu, Leyla Mollamahmutoğlu

**Affiliations:** H. Kalyoncu University, Health Science College, G Antep, Turkey; Zekai Tahir Burak (ZTB) Women’s Health Research and Education Hospital, Ankara, Turkey

**Keywords:** Postpartum depression, Psychosocial risk factors, Physical violence, Suicidal thoughts

## Abstract

**Background:**

Postpartum depression (PPD) is moderate to severe depression in a woman after she has given birth. Findings from several well-designed studies reflect great variability in rates, from 10 to 22%, and also in risk factors for PPD. This variability may reflect geographical location. The incidence and risk factors for PPD among Turkish women are not well documented. It is, however, important to understand the risk factors to develop preventive intervention strategies. This study aims to examine the prevalence of PPD and associated risk factors among a sample of women receiving services at a tertiary obstetrics hospital in Ankara, Turkey.

**Methods:**

A sample of 671 women, between 36 and 40 gestational weeks, were enrolled and screened for depressive symptomatology using the Hospital Depression Inventory. Sociodemographic and clinical data were also collected. At a subsequent postpartum evaluation, 6–8 weeks post-delivery, 540 of the 671 were screened using the Edinburgh Postnatal Depression Scale (EPDS) for PPD.

**Results:**

Eighty-three (15.4%) of the 540 women had scores above the cutoff point (>13) on the EPDS. Statistically significant correlations were found between antenatal, prenatal and postpartum depression scores (r = 0.24). Women reporting suicidal thoughts during pregnancy (OR: 6.99), history of past PPD (OR: 6.64), physical violence during pregnancy (OR: 6.20) or during the postpartum period (OR: 5.87), previous psychiatric history (OR: 4.16), depressive symptoms during pregnancy (OR: 1.70), subjectively lower level of satisfaction with the pregnancy (OR:0. 69), a history of premenstrual syndrome (PMS) (OR: 2.05), and unplanned pregnancy (OR: 1.69) had higher odds for developing PPD.

**Conclusion:**

One in six mothers screened as positive for PPD. Women who had previously been diagnosed with PPD, reported suicidal thoughts during pregnancy, or had been exposed to physical violence were at especially high risk for postpartum depression. To prevent and treat postpartum depression, special attention should be paid to women reporting these characteristics.

## Background

Postpartum depression (PPD) is a term applied to depressive symptoms occurring during the postpartum period. According to previous studies, PPD is the most common complication of childbearing [1] Symptom patterns in women with PPD are similar to those seen in women with depression unrelated to childbirth [2]. A depressed mother may not develop a positive and satisfying relationship with her infant to offset the stresses of newborn care and postpartum recovery and this may continue to affect children into toddlerhood, the preschool years and beyond [3]. Because of the potential for these serious consequences, mothers at risk of developing PPD need to be identified early, preferably during pregnancy, or at the latest immediately after delivery [4].

The precise level of incidence of PPD is uncertain. The reported rate ranges from 10 to 22% of women and seems to depend on the assessment method used, the timing of the assessment, and the cultural characteristics of the population [5]. Many risk factors have been identified for the condition and it is particularly tempting to attribute it to hormonal decline. Several other factors, however, may also predispose women to this condition. Previous studies have reported previous nonpuerperal depression, previous premenstrual dysphoria, stressful life events during pregnancy or early puerperium [6], poor social support [7], marital conflict [8] and violence [9], low levels of partner support [10], personality disorders [11], low income, immigrant status [12], young maternal age [13], obstetrical stressors, and difficult infant temperament [14] as predictors of PPD. The evidence is mixed for some factors, such as unwanted pregnancy [15] and gender of the newborn [16,17]. The likelihood of PPD does not appear to be related to the woman’s educational level, whether or not she breast-feeds, or the mode of delivery [17].

The aim of this study was to identify the prevalence and risk factors for PPD in a tertiary obstetric and gynecological hospital population, as reflected in a sample of Turkish women. There have been four large studies published so far on PPD prevalence in Turkey [18-21]. These studies found that the rate of PPD there was between 14% and 40.4%. Each of these four epidemiological studies relied on samples from one specific location, giving them some limitations. The difference between rates in different studies may relate to the samples and methods used. Turkish people show both Western and Eastern cultural characteristics depending on geographical location, and so the location of a study in Turkey may affect the rates of PPD identified. Previous studies found that PPD was more prevalent in the Eastern part of Turkey [22,23]. Ankara is the capital, and the second largest city in Turkey, with a population of almost 5 million in 2015. Its population shows a good mixture of typical psychosocial features. This research was carried out in the largest obstetric hospital in Ankara, with almost 30.000 deliveries per year. It acts as a tertiary referral center for patients both from around Ankara and from across Turkey, and therefore some of its patients are partly to be representative of the different geographical parts of the country. To our knowledge, this is the first study that has been conducted in a tertiary obstetrics and gynecology hospital in Turkey.

The risk factors assessed included the sociodemographic and clinical characteristics of the subjects, such as prenatal depression level, prenatal and postnatal history of physical abuse or exposure to intra-familial violence, gender of the newborn, experience of the pregnancy, whether the pregnancy was planned or unplanned, and previous psychiatric history of the subjects and their families.

The study hypotheses were as follows:

1. PPD is a common disorder in Turkish women.
2. Women who have marital problems (e.g., domestic violence, living in unfavorable extended family conditions), a negative view of the pregnancy (e.g., unplanned or unwanted pregnancy) or a previous psychiatric history are at significantly higher risk of developing PPD.

## Methods

This prospective study was conducted between January and July 2008 in Dr. Zekai Tahir Burak Women’s Health Research and Education Hospital which is a specialized tertiary obstetrics and gynecology hospital in Ankara, Turkey.

The study was approved by the Ethics Committee of the Dr. Zekai Tahir Burak Women’s Health Research and Education Hospital. Participants provided signed informed consent, after being told about the method and purpose of the study.

Any women with a gestational age of 36–40 weeks, and who could speak, read and write in Turkish, were eligible for inclusion. Women with multi-fetal pregnancies, a current or lifetime history of schizophrenia, a major chronic disease, or obstetric and pregnancy complications (severe preeclampsia or eclampsia, placenta previa, placental abruption, or major postpartum infection) were excluded, because these conditions might decrease the reliability of the assessment of PPD [24].

### Instruments

A total of 671 married women were evaluated at 36–40 gestational weeks using the Hospital Anxiety and Depression Scale (HADS) [25]. Seven items of the HADS relate to anxiety (HADS-A) and seven to depression (HADS-D). The HADS-D is a suitable instrument for assessing the level of depressive symptoms in pregnant women because it does not include physical indicators of psychological distress (such as headache or weight loss) that could be related to pregnancy and therefore lead to false positive results [26]. According to a validation study of the Turkish language version of HADS-D, the cutoff point for this scale is seven in a Turkish population [27].

The study used the Edinburgh Postnatal Depression Scale (EPDS), a 10-item questionnaire that is easy to administer and is an effective screening tool, to identify symptoms of PPD [28]. The reliability and validity of the Turkish version of the EPDS in identifying depression has been demonstrated [29] and it has been applied widely in both research and clinical settings. A score of 13 or higher has been used as the cutoff for PPD with the Turkish version. Evaluation with the EPDS was completed at 6–8 weeks postpartum. All of the subjects were invited to be assessed using the EPDS, and 540 of them (80.4%) took up the invitation.

The sociodemographic and clinical features of the patients were assessed using two structured self-report questionnaires developed by the researchers. The first, used prenatally, assessed both sociodemographic and clinical features. The second, used postnatally, focused only on clinical issues. The items on the questionnaire were designed to identify and assess the income level of the family, the occupation of the pregnant woman and her husband, and their education levels, family type (nuclear or extended), whether the pregnancy was planned or unwanted, attitudes towards the pregnancy and the baby, the presence of domestic violence, and previous psychiatric and obstetric history. Physical violence in the prepartum and postpartum period was assessed through interviews with the study subjects. The questionnaires also measured the level of satisfaction with the pregnancy on a Likert scale. The outcome of the pregnancy, the delivery method, and gender and health status of the baby were obtained from hospital records.

### Statistical methods

Statistical evaluation was carried out using SPSS for Windows. The Shapiro-Wilk test was used to test the normal distribution of continuous data. If the normality assumption for the comparison of means between two groups was satisfied, a Student’s *t*-test was used. If there was evidence of non-normality, the Mann–Whitney *U* test was used.

Frequencies and percentages for categorical data, and mean, standard deviations and median for continuous data were computed. Chi-square tests were used to examine the relationship between depression scores (dichotomized as 0 = <13, 1 = >13) and other categorical variables. Proportional comparisons were performed using Pearson’s Chi-square tests or Fisher’s exact test. Bivariate logistic regression analysis was used to evaluate the effects of other categorical variables on depression. A Pearson correlation analysis was used to assess predictor variables, and a linear regression analysis was performed to show the relationship between antepartum and post-partum levels of depression. All p-values reported were two-tailed, and the statistical significance limit was set at 0.05.

## Results

The mean age of the study group was 26.12 ± 5.15. Most of the participants were stay-at-home mothers (n = 480, 88.9%) and most came from middle and lower income families (80.8%). The mean education level of the study group was 8.56 years. Most of the pregnancies were planned (80.1%).

The rate of antepartum depression for the study group, according to the hospital depression and anxiety scale was 192 out of 540 (31.1%). In the EPDS postpartum evaluation of depression levels, 83 women (15.4%) showed scores above the cutoff point of 13 (Table 1). For ease of reference, this group is described as having PPD, even though no formal diagnosis was made in many cases.

**Table 1:** **Socio-demographic and clinical characteristics of the sample (n = 540)**

	**PPD group (EPDS ≥ 13) n = 83**	**No PPD (EPDS < 13) n = 457**	**t/** ***χ*** **2 value**	**Significance P**
Age (years) Mean ± SD	25.4 (±5.60)	26.26 (±5.05)	1.35	0.17
Duration of Education (year)	8.13 (±3.05)	8.64 (±3.30)	1.30	0,19
Duration of Marriage	5.69 (5.28)	5.52 (4.76)	−0.27	0,78
Family type				
Nuclear family n (%)	53 (14.7)	307 (85.3)	0.37	0.54
Extended family n (%)	29 (16.8)	144 (83.2)		0.52-1.40
Income				
Lower	28 (18.2)	126 (81.8)	3.96	0.13
Moderate	40 (15.3)	221 (84.7)		
Higher	9 (9.1)	90 (90.9)		
Employment status, n (%)				
Employed	4 (6.7)	56 (93.3)	3.93	0,03*
Housewife	79 (16.5)	401 (83.5)		0.97-7.82
Number of children				
0	39 (14.1)	238 (85.9)	3.27	0.35
1	25 (15.3)	138 (84.7)		
2	16 (22.5)	55 (77.5)		
3	3 (12.5)	21 (87.5)		

*Abbreviations*: *PPD* Postpartum depression, *SD* Standard Deviation, *EPDS* Edinburgh Postnatal Depression Scale *: statistically significant p < 0.05.

The sociodemographic and clinical characteristics of the groups scoring less and more than 13 in the EPDS evaluation are shown in Table 1. The PPD rate was higher in stay-at-home mothers than in women in paid employment (16.5% vs 6.7%; relative risk (RR) = 2.76, confidence interval (CI) 0.97–7.82, p = 0.057). The rate was also higher in women with an unplanned or unwanted pregnancy than in women whose pregnancy had been planned (21.5% vs 14.0%; RR = 1.69, CI 0.99–2.88, p = 0.05). Women with PPD also showed significantly more dissatisfaction with their pregnancy (P < 0.001) on a subjective level.

The clinical features of the two groups were compared and a binary logistic regression analysis was performed. Women with a previous history of a psychiatric disorder showed a higher PPD rate than those without such a history (RR = 4.16, CI 1.72–10.05, p = 0.001). The PPD rate was also higher in women who had experienced previous PPD episodes (RR = 6.64, CI 2.11–20.81, p = 0.001).

**Figure 1:**
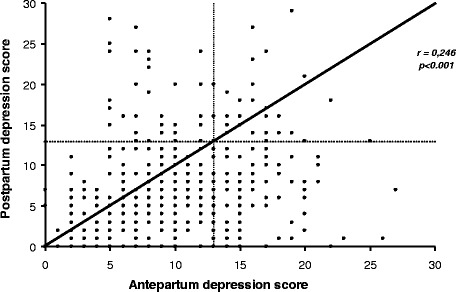
Relationship between antepartum and postpartum depression scores as measured by the HADS-D and EPDS.

Prenatal depression scores, as measured by the HADS-D, were higher for women who later developed PPD (11.97 vs 9.64, p < 0.0001). A correlation analysis showed a significant relationship between antepartum depression scores, as measured using the HADS-D, and PPD scores, as measured by the EPDS (PPD score = 4.339 + 0.287*antepartum depression score, r = 0.246, P = 0.001) (Figure 1).

The regression analysis showed that suicidal thoughts during pregnancy were the risk factor most strongly related to PPD (odds ratio 6.99, CI 2.08–23.49). Two other factors were also strongly related: a history of premenstrual syndrome and exposure to physical violence during pregnancy or the postnatal period. Women with PMS had rates of PPD of 21.1%, while the rate for other women was 11.7% (RR = 2.049, CI 1.25–3.33, p = 0.003). Women who had been exposed to domestic physical violence during the prepartum period (50%, RR = 6.20, CI 2.49–15.45, p = 0.001) and also during the postpartum period (50%, RR = 5.87, CI 2.00–17.20, p = 0.001) both showed higher levels of PPD.

No differences were found in the PPD rate for women who experienced an early infant death and those who did not (Table 2). Likewise, there were no significant differences for other sociodemographic and clinical factors, such as the years of education or income level of the mother, the number of previous children, the family type (extended or nuclear), hyperemesis during pregnancy, abnormalities in the newborn, satisfaction with the gender of the newborn, the health status of the newborn, or breast-feeding status.

**Table 2:** **Comparison of marital, social and psychological risk factors between women with and without postpartum depression**

	**PPD group (EPDS ≥ 13)**	**No PPD (EPDS < 13)**	**t/** ***χ*** **2 Value**	**P value**	**Relative Risk Odds ratio**	**CI**
**Prenatal depression Score** ^**a**^	11.97 (4,98)	9.64 (4.70)	−3.57	0,000*	1,70	0,99-2,90
**Satisfaction level about the pregnancy period** ^**b**^	2.28(1.24)	2.81(1.17)	3.80	0.000*	0,69	0,57-0,84
**Suicidal Thoughts in Pregnancy**						
Yes	6 (54.5)	5 (45.5)	13.13	0,003*	6.99	2.08-23.49
No	77 (14.6)	449 (85.4)				
**Hyperemesis n (%)**						
Yes	5 (10.4)	43(89.6)	0,43	0.32	0,62	0.23-1.60
No	75(15.9)	397(84.1)				
**Planned pregnancy n (%)**						
Yes	60 (14)	370 (86)	3,72	0.05*	1,69	0.98-2.88
No	23 (21.5)	84 (78.5)				
**Anomaly in Newborn n (%)**						
Yes	2 (13.3)	13 (86.7)	0,04	0,83	0,85	0,19-3,85
No	80 (15.3)	444 (84.7)				
**Satisfaction with the gender n (%)**						
Yes	57 (14.7)	348 (85.9)	1,23	0.26	1,37	0.78-2.40
No	20 (18.3)	220 (84.9)				
**Violence in pregnancy n (%)**						
Yes	10 (50)	10 (50)	19,46	0.001*	6.20	2.49-15.45
No	71 (13.9)	441 (86.1)				
**Postpartum Violence n (%)**						
Yes	7 (50)	7 (50)	13,08	0.001*	5.87	2.00-17.20
No	76 (14.6)	446 (85.4)				
**Past PPD History**						
Yes	6 (54.5)	5(46.5)	13,41	0.001*	6.64	2.11-20.81
No	39 (14.4)	222 (85.6)				
**Past Psychiatric History**						
Yes	9(39.1)	14 (60.9)	11,63	0.001*	4.16	1.72-10.05
No	75 (15.9)	389 (86.6)				
**PMS History**						
Yes	39(21.1)	144 (78.7)	8,56	0.003*	2.05	1.25-3.33
No	39 (11.7)	295 (88.3)				
**Infant Death**						
Alive	82(15.4)	451(86.1)	0,706	0.70	0,917	0.109-7.71
Death	1(14.3)	6(85.7)				
**Breast Feeding**						
Yes	78 (15)	442 (85)	0,090	0,48	1.21	0,34-4,32
No	3 (17.6)	14 (82.4)				

^a^As measured by Hospital Depression Scale; ^b^in a Likert type scale; 1- ‘not satisfied at all’ to 5- ‘very satisfied’ *: statistically significant p < 0.05. *Abbreviations*: *CI* Confidence Interval, *PPD* Postpartum depression, *SD* Standard Deviation, *EPDS* Edinburgh Postnatal Depression Scale.

## Discussion

The overall PPD rate of 15.4% found in this study is thought to reflect the overall rate for the women admitted to this hospital. According to studies conducted in Western countries, the incidence of PPD is approximately 10–15% of all mothers [30,31]. Findings from previous large and well-designed studies in Western countries reflect high variability in the estimates of PPD. In non-Western countries, reported prevalence rates also vary widely, from 16% in Zimbabwe through 22% in Jordan [32], to 34.7% in South Africa [33], and 6–25% in India [34]. In Turkey, the rate of PPD has been reported to be between 14 and 40.4% [16-19]. The rate in this study is consistent with that found by Danaci et. al [18]. They carried out their study in different primary health care centers in Manisa, a city in the western part of Turkey, and found a rate of 14% [20]. Another study in the north part of Turkey, in the city of Trabzon, found a rate of 28.1%. The highest rate found in Turkey was 40.1 %, in a study carried out in Edirne, a west border city, which used a very small sample [19]. This difference between rates in the same country, as previously discussed, may relate to the study samples and methods, and also the geographical location.

Previous studies about PPD have identified several related risk factors. This study found that stay-at-home mothers, women exposed to violence during pregnancy or the postpartum period, or who have a history of PMS, unplanned pregnancies, previous psychiatric history (particularly previous depressive episodes), depressive symptoms, or suicidal thoughts in pregnancy, have higher rates of PPD than other women. The factors most strongly related to PPD were suicidal thoughts, past history of PPD, and domestic violence. Other significant factors were previous psychiatric history, depressive symptoms during pregnancy, a history of PMS, unplanned pregnancies and dissatisfaction with the pregnancy.

Confirming previous studies [35], this study found that depression during pregnancy and a previous history of PPD were strongly related to development of PPD. There was also a strong correlation between PPD and antepartum depressive symptoms, as measured by the HADS-D. Suicidal thoughts during pregnancy were the risk factor most strongly related to PPD (odds ratio 6.99, CI 2.08–23.49). Suicidal thoughts can of course be a symptom of depression, but these findings indicate that such thoughts may be the most important depressive symptom for predicting PPD. Although suicidal thoughts was an important indicator for severity of depression in Turkish population [36], the literature review failed to identify any other study that particularly focused on the relationship between antepartum suicidal thoughts and PPD.

Many studies have linked physical violence during pregnancy with PPD [10,37], which is consistent with the findings of this study. One study from China [38] focused on domestic violence, and reported that domestic violence during pregnancy was an important contributing factor for PPD. An extensive review found that pooled estimates from longitudinal studies suggested a three-fold increase in the likelihood of high levels of depressive symptoms during the postnatal period when partner violence was reported during pregnancy (OR = 3.1, 95% CI 2.7–3.6) [39]. This study defined ‘violence’ as physical violence only, and the high odds ratio may be related to this narrow definition.

Other reported psychosocial and clinical risk factors for PPD include anxiety during pregnancy, previous nonpuerperal depression, previous premenstrual dysphoria, stressful life events during pregnancy or the early puerperium, poor social support, marital conflict [8], low levels of partner support [1], gender of the newborn [16], personality disorder, low income, immigrant status, and young maternal age. Risk factors shown to have more modest associations with PPD include low socioeconomic status, being single, an unwanted pregnancy [15], obstetrical stressors, and difficult infant temperament [14]. This study found that PPD was clearly related to PMS history, unplanned pregnancy, and past psychiatric history, and that women with PPD were significantly more dissatisfied with their pregnancy (P < 0.001). The PPD rate was also significantly higher in stay-at-home mothers. There were no differences related to any other factors.

This study has several limitations. First, the EPDS assesses symptoms and is used as a screening tool. A more complete clinical interview could have been used to establish a more reliable diagnosis of postnatal depression. Second, only physical violence was included in the assessments, and verbal or emotional abuse might also have an effect. Although our hospital is the biggest obstetric center of capital of Turkey, it has limitation of the single source (even if the hospital draws from across Turkey) – presumably because it is a tertiary centre, which could also bias the sample, the sample would mostly reflect the population of Ankara, not exactly the whole country. The strength of this study is that it is the first conducted in a tertiary obstetrics center in Turkey, and included most of the parameters previously reported to be linked to occurrence of PPD.

## Conclusion

With a prevalence rate of 15%, this study identifies PPD as an important issue in women’s reproductive health. By studying a tertiary obstetric hospital in the second largest city in Turkey, with patients drawn from both the immediate surroundings and more widely, the sample is more likely to reflect the characteristics of the Turkish population. Analysis of the clinical and sociodemographic data found that women who had previously experienced PPD, reported suicidal thoughts during pregnancy, or who had been exposed to domestic violence are at high risk of developing PPD. Other factors significantly related to PPD were a previous psychiatric history, unplanned pregnancy, and a history of premenstrual dysphoric syndrome (PMDS). Women who show these characteristics should be offered special care, such as family therapy, counseling, or psycho-education about depression, to prevent and treat possible depression. Another important implication of this study is the importance of antenatal evaluation of pregnant women for PPD. The data suggest that antepartum depression scores strongly relate to PPD. By screening women antenatally, it may be possible to intervene early and treat or even prevent PPD.

## Abbreviations

PPD: Postpartum depression
SD: Standard deviation
EPDS: Edinburgh Postnatal Depression Scale
HADS-D: Hospital Depression Scale
CI: Confidence interval
